# Housing the Nursing Staffs

**Published:** 1921-01-01

**Authors:** Gladys M. E. Leigh


*To the Editor of* THE HOSPITAL.

### Housing the Nursing Staffs.

*To the Editor of* THE HOSPITAL.

SIR,—It is now no longer considered permissible to exploit one section of the community for the benefit of another, and the fact that the Committee of the North Staffs Cripples' Hospital were sufficiently fortunate to find nurses willing and able to run the risk of impairing their health rather than leave helpless cripples without skilled attention does not relieve the Committee of its legitimate obligations. Have the governors of the hospital and their friends no spare rooms in their houses which they could lend to the institution as temporary accommodation for the nursing staff during two short months of inclement weather? Nowadays we hear continually of sacrifice and service, but it is time that hospital committees and the general public understood *clearly* that these attributes are by no means peculiar to the nursing services, but may be shared by all.

It would be well for the Rector of Stoke and his parishioners to remember "the labourer is worthy of his hire."—Yours faithfully, GLADYS M. E. LEIGH.

